# Indications, clinical outcomes and complications of 1,949 flexible bronchoscopies

**DOI:** 10.31744/einstein_journal/2018AO4380

**Published:** 2018-10-30

**Authors:** Altair da Silva Costa, Paulo Rogerio Scordamaglio, Iunis Suzuki, Addy Lidvina Mejia Palomino, Marcia Jacomelli

**Affiliations:** 1 Hospital Israelita Albert Einstein, São Paulo, SP, Brazil.

**Keywords:** Bronchoscopy, Critical care, Respiratory tract infection, Broncoscopia, Cuidados críticos, Infecções respiratórias

## Abstract

**Objective:**

To describe indications, clinical outcomes and complications of flexible bronchoscopy.

**Methods:**

A descriptive observational study of bronchoscopies performed at the endoscopy service of *Hospital Israelita Albert Einstein* . Demographic (age, gender and origin) and medical (indications and results of endoscopy and diagnostic tests, such as biopsy collection, lavage, cytology and culture) data were analyzed. Electronic medical records with incomplete data or reporting interventional procedures were excluded.

**Results:**

Over a three-year period (2013 to 2016), a total of 1,949 bronchoscopies were performed by respiratory endoscopy team and anesthesia specialists of the hospital. The mean age of patients was 57.7±21.9 years (range of 3 days to 99 years), with prevalence of males (56.4%). The procedures were mostly (86.3%) elective and 30.7% were carried out in the intensive care unit. Major indications for bronchoscopy were infection or secretion (42.4%), followed by suspected neoplasm (10.8%). Endoscopic changes were reported in 91.9% of cases, with more than one change described in approximately 6.9% of patients. Positive results were obtained via direct testing or culture in 36.3% and 53.9% of 1,399 bronchoalveolar lavages, respectively. The overall diagnostic yield (bronchoalveolar lavage and biopsy) was 72.6%. Mild adverse event rate was 7.2%. The rate of severe adverse events requiring additional intervention was 0.5% (pneumothorax, 0.4%; severe bleeding with patient death, 0.1%).

**Conclusion:**

Lower airway endoscopy is critical for respiratory disease assessment, diagnosis and treatment. Flexible bronchoscopy is associated with good diagnostic yield and minimal inherent risk.

## RESUMO

Objetivo Descrever as indicações, os resultados clínicos e as complicações associadas à broncoscopia flexível. Métodos Foi realizado um estudo observacional descritivo das broncoscopias realizadas no serviço de endoscopia do Hospital Albert Einstein. Foram analisados: informações demográficas, como idade, gênero e procedência; dados clínicos sobre a indicação do exame; e resultados endoscópicos e dos exames diagnósticos realizados, como biópsias, lavados, citologias e culturas. Os fatores de exclusão foram dados incompletos no sistema e procedimentos intervencionistas. Resultados No período de 3 anos, de 2013 a 2016, foram realizadas 1.949 broncoscopias no hospital pela equipe da endoscopia respiratória e anestesiologista. A média de idade dos pacientes foi de 57,7±21,9 anos, (variação: 3 dias a 99 anos), com prevalência do gênero masculino (56,4%). A maioria dos exames (86,3%) foi eletiva e 30,7% foram realizados na terapia intensiva. A indicação médica mais frequente para realização da broncoscopia foi infecção ou secreção (42,4%), seguida de suspeita de neoplasia (10,8%). Nas alterações endoscópicas das broncoscopias realizadas, obtivemos os dados em 91,9% dos exames. Cerca de 6,9% dos pacientes apresentaram mais de uma alteração endoscópica. Foram realizados 1.399 lavados, com positividade de 36,3% nas pesquisas diretas e 53,9% nas culturas. O rendimento geral com lavados broncoalveolares e biópsia foi de 72,6%. Em nossa série, a taxa de eventos adversos leves foi de 7,2%. Os eventos adversos graves, nos quais foi necessária alguma intervenção adicional, somaram 0,5%: 0,4% de pneumotórax e 0,1% de hemorragia grave com óbito. Conclusão A endoscopia das vias aéreas inferiores é imprescindível para avaliação, diagnóstico e tratamento de doenças respiratórias. A broncoscopia flexível tem bom rendimento diagnóstico e risco mínimo associado.

## INTRODUCTION

Respiratory endoscopy was revolutionized by the invention of the flexible bronchoscope by Dr. Ikeda, in 1968. At first, bronchoscopy was performed using a rigid instrument in highly specialized centers.^(^ ^1^ ^)^ As from 1970, the popularization of flexible bronchoscopy over rigid bronchoscopy led to a rise in the number of procedures performed and increasing availability at hospitals and outpatient clinics. Flexible bronchoscopy, a highly versatile and effective diagnostic and therapeutic tool, plays a key role in respiratory medicine.^(^ ^1^ ^,^ ^2^ ^)^ Lower airway endoscopy is aimed at anatomical assessment for diagnostic and therapeutic purposes.

Major indications are infectious conditions, such as pneumonia, bronchitis and bronchiectasis. Bronchoscopy is useful for clearance of airway secretions, relief of related airway obstructions, for diagnosis and identification of pathogens. Other indications for respiratory endoscopy comprise thoracic neoplasm, hemoptysis, airway management ( *i.e* ., cannula replacement and intubation), foreign body retrieval, bronchial stenosis, and transplantation.^(^ ^1^ ^,^ ^3^ ^)^

The scope of indications for interventional bronchoscopy currently includes tracheal or bronchial obstruction relief, mediastinal staging, dilation and others. Bronchoscopy has also experienced major advances with the advent of novel technologies, such as endoscopic laser resection, deployment of endobronchial orthoses devices, brachytherapy catheters, electrocauterization techniques aimed at hemostasis or tissue resection, therapeutic and diagnostic cryotherapy, endobronchial ultrasound (EBUS) and electromagnetic scanning, among others.^(^ ^2^ ^,^ ^4^ ^,^ ^5^ ^)^

Limited availability is still a significant constraint for application of these novel technologies in Brazil. Also, national respiratory endoscopy data are lacking and the number of expert professionals is low.^(^ ^3^ ^)^

## OBJECTIVE

To describe major indications, outcomes and complications of flexible bronchoscopy.

## METHODS

An observational descriptive study based on a prospective database of bronchoscopies carried out at the endoscopy service of *Hospital Israelita Albert Einstein* , in São Paulo (SP). Data compiled over a 36-month period (3 years), from April 2013 to April 2016, were analyzed.

The sample comprised bronchoscopic procedures carried out at the endoscopy service of *Hospital Albert Einstei* n – Morumbi unit. Electronic medical records with incomplete data or reporting interventional procedures, such as tumor resection, endoscopic ultrasound and airway dilation, were excluded.

Demographic (age, gender and origin) and medical (indications and results of endoscopic and diagnostic procedures, such biopsy collection, lavage, cytology and culture) data were also investigated.

This study was limited to descriptive analysis and did not include inferential statistics. The Ethics Committee of *Hospital Israelita Albert Einstein* approved this study; CAAE: 52243515.4.0000.0071.

## RESULTS

The total of 1,949 bronchoscopies were performed over the course of three years (2013 to 2016), by the respiratory endoscopy team of the hospital. Anesthesiologists, in compliance with the institutional protocol, assisted the procedures. Anesthetic techniques ranged from sedation with spontaneous breathing to general anesthesia delivered via endotracheal intubation or laryngeal mask. The mean age was 57.7 years (standard deviation, 21.9 years; median, 62 years; age range, 3 days to 99 years). This sample comprised 1,100 (56.4%) male patients.

Bronchoscopic procedures involved intensive care unit (ICU) patients, outpatients or inpatients (30.7%, 27.1% and 23.1% cases respectively).

**Figure 1:**
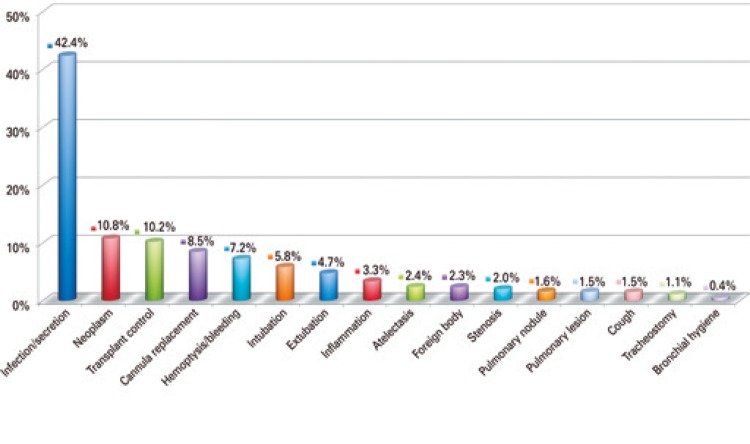
Medical indications for bronchoscopy

Most procedures (86.3%) were elective, with only 13.7% of emergency bronchoscopies. Medical indications were reported in 80% of cases. The most frequent indication for bronchoscopy was suspected infection or secretion (42.4%), followed by suspected neoplasm (10.8%). More than one indication for bronchoscopy was reported in 5.7% cases ( Figure 1 ).

We were able to report endoscopic alterations in 91.9% of cases, with more than one described in 6.9% of patients. Upper airway changes were described in 5.5% of cases; in that, laryngitis (4.4%) and tumor lesions, such as polyps, cysts or nodules (0.7%). Bronchoscopy was normal in most patients in this sample (21.3%). Secretion was the second most common finding reported (17.4%) ( Table 1 ).

**Table 1:** Endoscopic changes in analyzed bronchoscopies

Endoscopic changes	n (%)
Endoscopic results analyzed	1,792 (91.9)
Not informed/not available result	157 (8.1)
No changes (normal)	382 (21.3)
Secretion	312 (17.4)
Transplant control	159 (8.9)
Cannula replacement	132 (7.4)
Endobronchial lesion	104 (5.8)
Tracheobronchitis	95 (5.3)
Hemoptysis	92 (5.1)
Intubation	91 (5.1)
Bronchitis	73 (4.1)
Secretion plug	61 (3.4)
Purulent bronchitis	48 (2.7)
Foreign body	37 (2.1)
Dynamic airway collapse	36 (2.0)
Stenosis	32 (1.8)
Difficult airway	25 (1.4)
Tracheomalacia	25 (1.4)
Clots	21 (1.2)
Extrinsec compression	20 (1.1)
Malformation/anatomical variation	12 (0.7)
Bronchoaspiration	10 (0.6)
Tracheal rupture	10 (0.6)
Granuloma	8 (0.4)
Anastomosis dehiscence	5 (0.3)
Bronchopleural fistula	2 (0.1)

Ancillary tests requested were also analyzed (biopsy, bronchoalveolar lavage (BAL), culture and others). Bronchoscopic BAL was performed in 71.8% (n=1,399) of patients; positive results were obtained via direct testing or culture in 36.3% and 53.9% of cases, respectively. Biopsies were collected from 37.6% of patients. Overall diagnostic yield, including ancillary tests (BAL and biopsy), amounted to 72.6%. Specific tests are shown in table 2 .

**Table 2:** Tests performed in bronchoscopies

Test	Performed (n)	Positive BAL (n)	Positive results of tests in relation to methods (%)
BAL	1,399		
Direct testing − microorganisms	932	338	36.3
Culture for aerobes	923	482	52.2
MTB test	899	12	1.3
MTB culture	849	23	2.7
Fungal culture	1,159	242	20.9
Oncotic cytology	425	64	15.1
Polymerase chain reaction	388	83	21.4

BAL: bronchoalveolar lavages; MTB: Mycobacterium tuberculosis bacillus.

Major microorganisms detected by different BAL techniques are described in table 3 . Mycobacterial or respiratory viral infections were diagnosed in 35 and 33 patients (2.5% and 2.3% of 1,399 BALs, respectively). Respiratory viruses detected via direct testing were H1N1, rhinovirus, *Parainfluenzae* and s *yncytial* virus t *ype* B (six, four, two and two cases, respectively). The following viruses were detected by polymerase chain reaction (PCR) analysis: syncytial virus (five cases), herpes virus type 1 (five cases), rhinovirus (three cases) and s *yncytial* virus type A (three cases; two *influenzae* and one *adenoviru* s) *.*

**Table 3:** Microorganisms most frequently identified in each bronchoalveolar lavage method

Methods and microorganism found	Numbers
Direct testing	
Gram positive	153
Gram negative	40
*Aspergillus galactomannan*	36
Rare forms budding yeast	24
Respiratory virus	14
MTB test	12
*Legionella*	1
Aerobes culture	
*Estreptococos viridans*	217
*Pseudomonas aeruginosa*	65
*Candida albicans*	36
*Klebsiella pneumoniae*	27
*Estafilococos aureus*	25
MTB culture	
*Mycobacterium tuberculosis*	16
*Mycobacterium avium*	4
*Mycobacterium kansasii*	3
Fungal culture	
*Candida albicans*	167
*Candidas glabrata*	20
Polymerase chain reaction	
Cytomegalovirus	53
Respiratory virus	19
*Pneumocystis jirovecii*	8

MTB: Mycobacterium tuberculosis bacillus.

Ancillary procedures performed were as follows: 732 biopsies (37.2% of 1,949 bronchoscopies), 132 cannula replacements and 91 endoscopic intubations. Biopsy diagnostic yield corresponded to 49%. Among the biopsies performed, the neoplasms were diagnosed in 114 (15.6%) patients; pulmonary adenocarcinoma was the most common (49 patients, 6.7%) ( Table 4 ). Other pathological diagnoses were absence of rejection (67 patients, 9.1%), inflammatory process or chronic infiltrate (31 patients, 4.2%), acute rejection (25 patients, 3.4%), bronchiolitis (21 patients, 2.9%), organizing pneumonia (17 patients, 2.3%), subacute pulmonary injury (17 patients, 2.3%) and granulomatous process (15 patients, 2.0%) of the biopsies performed.

**Table 4:** Only biopsies with neoplasms, total of 114 tests

Neoplasm	n (%)
Adenocarcinoma	49 (43.0)
Non-small cell carcinoma	15 (13.2)
Epidermoid carcinoma	12 (10.5)
Small cell carcinoma	11 (9.6)
Carcinoid tumor	10 (8.8)
Undifferentiated carcinoma	8 (7.0)
Sarcoma	2 (1.8)
Hamartoma	3 (2.6)
Others	4 (3.5)

The rate of complication was 7.2% (141 adverse events in 1,949 procedures). The most commonly reported were mild and moderate adverse events as hypoxemia (oxygen saturation level less than 85%) in 4.9% of bronchoscopies, and bleeding, in 2.1%. Severe adverse events requiring additional intervention were reported in ten cases in this sample (pneumothorax, 8 cases, 0.4%; severe bleeding leading to patient death, 2 cases; 0.1%).

## DISCUSSION

Bronchoscopy is a vital, well-established tool with rising attractiveness in respiratory medicine. Bronchoscopy must be properly indicated and performed in a safe and effective manner; still, it is an operator-dependent procedure and outcomes vary according to different methods and techniques.^(^ ^2^ ^,^ ^4^ ^,^ ^6^ ^)^ More than one endoscopy specialist may be required in specific, exceptional situations.

In other services, sedation may be performed by certified endoscopy specialists. Scientific evidence supporting the need for anesthesiologist participation is limited and derived from non-analytical studies ( *i.e* ., case reports, case series or specialist opinions).^(^ ^2^ ^,^ ^4^ ^,^ ^7^ ^)^ Our institutional protocol directives that bronchoscopy must be carried out by at least two physicians – one respiratory endoscopy and one anesthesiologist. Topical anesthesia with 1% lidocaine is required to help control coughing, and reduces sedating drug doses. High-standard randomized trials suggest lower lidocaine concentrations are as effective as higher concentrations for cough control during bronchoscopy (level 1 evidence). Topical nasal anesthesia is thought to be more effective when 2% lidocaine gel is used (grade A recommendation).^(^ ^2^ ^)^

The demographic profile of patients in this sample (prevalence of male patients aged 50 to 60 years) is consistent with literature data.^(^ ^2^ ^,^ ^4^ ^,^ ^5^ ^)^ Age *per se* is not a risk factor or contraindication for bronchoscopy.^(^ ^2^ ^)^ Most patients (30.7%) in this study were at an intensive care unit, the hospital sector where respiratory endoscopy is more often performed, with 23% of ICU patients required bronchoscopy at some point.^(^ ^6^ ^-^ ^8^ ^)^

Major indications for bronchoscopy in ICU were infection, obstruction by secretion and atelectasis.^(^ ^2^ ^,^ ^6^ ^-^ ^8^ ^)^ Bronchoscopic BAL for infection diagnosis in patients receiving mechanical ventilation is associated with oxygenation compromise and reduced oxygen partial pressure/inspired oxygen fraction (PaO_2_/FiO_2_) ratio, regardless of BAL fluid volume.^(^ ^2^ ^)^ Therefore, physicians must be aware of contraindications for bronchoscopy to minimize severe complications, such as hemodynamic instability, bronchospasm, severe unresponsive hypoxemia and hemorrhage. Overall, bronchoscopy is a safe procedure, when the safety precautions are taken.

Bronchoscopy requires special care in ICU settings, particularly in patients receiving mechanical ventilation. Bronchoscope diameter must be consistent with endotracheal tube diameter to prevent significant airflow obstruction and increased airway resistance. In non-intubated patients, the bronchoscope occupies 10 to 15% of the tracheal cross-sectional area, in contrast with 30 to 81% of the endotracheal tube cross-sectional area in intubated patients. Hence, poor scope choice may lead to respiratory comprise and interfere with aspiration of secretion and BAL collection. Endoscopists must be aware of the ratio between the bronchoscope and endotracheal tube diameter to ensure a safe, high-quality, risk-free procedure and prevent scope damage. Examples of proportions of tube cross-sectional area obstructed by different bronchoscopes are given in table 5 .^(^ ^1^ ^,^ ^2^ ^,^ ^6^ ^)^

**Table 5:** Diameters and areas of tracheal cannulae, and proportion of obstruction

	Tracheal cannula				
Inner bore, mm	7	7.5	8	8.5	9
Area, mm^2^	38.5	44.2	50.3	56.7	63.6
Cannula area ratio obstructed,%					
BF-P160*	49	43	38	33	30
BF-XT160^†^	81	71	62	55	49

* diameter: 4.9mm; área: 18.8mm^2^; ^†^ diameter: 6.3mm; area 31.2 mm^2^.

Most of the bronchoscopies showed endoscopic alteration, in 1,410 patients (78.7%), compatible with the consulted literature.^(^ ^1^ ^,^ ^3^ ^,^ ^5^ ^)^ In 382 patients (21.3%), the exams were normal but it does not mean without pulmonary alterations, refers only to the endoscopic features of the bronchial tree.

Mechanical constraints are dictated by scope diameter. The most usually used bronchoscope is 4.9mm wide. The bronchial tree is partitioned into approximately 20 generations of dichotomous branching, extending from the initial portion of the trachea to terminal bronchioles (20mm and 1mm wide, respectively). Therefore, bronchoscopic imaging is limited to the fourth/fifth generation of branches. Distal changes ( *i.e* ., beyond the sixth generation of branches) can only be assessed indirectly via ancillary endoscopic or imaging modalities, such as endobronchial ultrasound and radioscopy. For example: normal endoscopic findings in patients with pulmonary infiltrate or pneumonia requiring BAL or pulmonary biopsy, in the absence of endobronchial lesions.

Bronchoscopy is indicated for non-resolving or slowly resolving pneumonia in immunocompetent patients with suspected infection, particularly in smokers or former smokers aged over 50 years.^(^ ^2^ ^)^ Bronchoscopy is most often indicated to determine of the etiologic diagnosis in cases of infection or secretion.^(^ ^2^ ^,^ ^4^ ^)^ In 42.4% of patients in this sample, the bronchoscopy was indicated due to infection or secretion. Direct testing and culture were positive in 36.3% and 53.9% of cases, respectively. The sensitivity of microscopy ranges from 10 to 40%, compared to 52 to 95% of BAL culture.^(^ ^2^ ^)^ Some microorganisms ( *Candida* spp., *coagulase-negative Staphylococcus* , *Corynebacterium* spp., *Enterococcus* , *Neisseria* spp., *Streptococcus viridans* , and yeasts) are thought to be non-pathogenic commensals, and are not regarded as positive cultures.^(^ ^9^ ^)^ Bronchoalveolar lavage is a sensible and specific tool for etiologic agent identification in immunocompromised patients.^(^ ^2^ ^,^ ^4^ ^,^ ^5^ ^)^ The diagnostic yield of different methods is organism-specific. For example, BAL has 90% to 98% sensitivity for detection of *Pneumocystis jirovecii* in cases with no history of therapeutic or prophylactic antimicrobial therapy.^(^ ^2^ ^)^ Empirical antimicrobial therapy reduces BAL sensitivity to 64%. Bilateral BAL is associated with higher diagnostic yield compared to unilateral BAL, and samples collected from upper lobes are more sensitive compared to those collected from lower or middle lobes.^(^ ^2^ ^)^ Bronchoalveolar lavage is as sensitive as transbronchial biopsy, and has lower inherent risk. Therefore, BAL is the diagnostic test of choice in suspected cases of *P* . *jirovecii* infection.^(^ ^2^ ^,^ ^6^ ^)^

In immunocompromised patients, PCR is used in BAL for rapid diagnosis of tuberculosis, with 85.7% sensitivity and 90.9% specificity. This diagnostic strategy is useful for detection of *Legionella* , mycobacteria, fungi ( *aspergillus* ) and respiratory viruses. Samples for *Legionella* culture must be collected using distilled water to prevent inhibition of microbial growth; in all other tests and cultures, saline solution.^(^ ^2^ ^)^In immunocompromised, HIV-positive patients with pulmonary infection, BAL is thought to be associated with changes of diagnosis and antimicrobial therapy in 50% and 62% of cases, respectively.^(^ ^2^ ^,^ ^4^ ^,^ ^5^ ^)^ Immunocompetent and immunocompromised patients were not compared in this study; still, we believe data presented may help optimize BAL performance and results.

Another interesting finding in this study was the prevalence of mycobacterial and viral infections (2.5% and 2.3% of 1,399 BALs, respectively). Isolation of such agents, although uncommon in this study, suggests special attention should be given to bronchoscopy regarding the need for rooms or environments amenable to respiratory isolation prior to and after the procedure. These measures are thought to be important to avoid cross-infections between patients and for occupational safety.

Suspected neoplasm was the second (10.8%) more common indication for bronchoscopy. Respiratory endoscopy is admittedly useful for pulmonary neoplasm diagnosis and staging. Endoscopically visible endobronchial lesions have higher diagnostic yield as compared to peripheral lesions.^(^ ^2^ ^,^ ^5^ ^,^ ^10^ ^)^ According to recent evidences, the expected diagnostic yield of combined endoscopically visible lesions and bronchial biopsy amounts to 90%. A minimum of five samples are required to achieve 90% diagnostic likelihood in biopsies of visible tumors.^(^ ^1^ ^,^ ^2^ ^,^ ^4^ ^)^ Peripheral lesions are approached via combined bronchoscopic techniques, such as BAL, biopsy and fine needle aspiration. In these lesions, size and possibility of fluoroscopic visualization are the major determinants of diagnostic yield. Positive biopsy rates range from 14% to 50% for pulmonary nodules (≤3cm), and 46% to 80% for pulmonary masses (>3cm).^(^ ^1^ ^,^ ^2^ ^,^ ^11^ ^)^ Advanced techniques, such as radial probe endobronchial ultrasonography (radial EBUS) have led to significant diagnostic yield improvement in bronchoscopy of peripheral lesions (74% and 92% for nodules and masses, respectively).^(^ ^12^ ^)^ Overall diagnostic yield of bronchoscopic biopsies in this series achieved 49%, and it is consistent with literature data (<50%).^(^ ^2^ ^,^ ^4^ ^)^ In this series, the cumulative diagnostic yield of bronchoscopic procedures (BAL and biopsy) was 72.6%. The significance of combined bronchoscopy procedures for increased diagnostic yield varied according to medical indication and patient/disease characteristics.

Tachycardia, bradycardia, mild bleeding, bronchospasm, laryngospasm, cough, dyspnea, sore throat and hypoxemia are common adverse events of bronchoscopy, cough being the most common.^(^ ^2^ ^)^ Severe adverse events, such as cardiac arrhythmia requiring intervention, severe hemorrhage, myocardial infarction, pulmonary edema, pneumothorax and death are uncommon. Bleeding may be categorized as traces of blood (spontaneuos resolution without continuous suctioning); mild (spontaneuos resolution with continuous suctioning of blood from the airways); moderate (intubation of biopsied segment with the bronchoscope, and administration of adrenaline and ice-cold saline for hemostasis); or severe (placement of bronchial blocker or fibrin sealant; reanimation, blood transfusion, admission in ICU or death).

Adverse event rates ranged from 5% to 35%. Severe complications are expected in less than 1% of patients.^(^ ^2^ ^,^ ^4^ ^,^ ^5^ ^,^ ^11^ ^)^ The adverse event rate in this series corresponded to 7.2%, including two deaths (0.1%). Death cases are always worthy of revision and reflection. The indication in critical situations should always be discussed with the clinical staff and the bronchoscopist - both aware of the limits, risks and benefits of the procedure. Deaths in this study involved critically ill patients. One patient was in the semi-intensive care unit with endocarditis, septic shock, kidney failure requiring dialysis and diffuse pulmonary infiltrate. Biopsy and BAL were requested for diagnostic purposes. Bronchoscopic biopsy collection was carried out after discussing the case with the medical team. Severe bleeding occurred upon collection of the first fragment, which was difficult to control and followed by severe hypoxia. The patient was admitted to the ICU following bleeding resolution and overall stabilization, but progressed to cardiac arrest and death. The second patient suffered from severe leukemia with compromised coagulation function and hemoptysis. Bronchoscopy was indicated for bleeding control, but hypoxia progressed during the procedure. Hemoptysis was temporarily controlled; however, hypoxia persisted and the patient died in a few hours.

In this study, the rate of adverse events corresponded to 0.5%, with 0.1% mortality. Therefore, bronchoscopy may be considered a safe procedure, provided proper techniques are used and contraindications accounted for.

## CONCLUSION

Lower airway endoscopy is a useful, critical tool for assessment, diagnosis and treatment of several respiratory diseases. Flexible bronchoscopy should be made widely available, given the good diagnostic yield and minimal inherent risk associated with the procedure when carried out by expert professionals.
